# Whole Genome and Exome Sequencing of Monozygotic Twins with Trisomy 21, Discordant for a Congenital Heart Defect and Epilepsy

**DOI:** 10.1371/journal.pone.0100191

**Published:** 2014-06-20

**Authors:** Pongsathorn Chaiyasap, Supasak Kulawonganunchai, Chalurmpon Srichomthong, Sissades Tongsima, Kanya Suphapeetiporn, Vorasuk Shotelersuk

**Affiliations:** 1 Interdepartment of Biomedical Sciences, Faculty of Graduate School, Chulalongkorn University, Bangkok, Thailand; 2 Genome Institute, National Center for Genetic Engineering and Biotechnology, Khlong Nueng, Khlong Luang, Pathum Thani, Thailand; 3 Center of Excellence for Medical Genetics, Department of Pediatrics, Faculty of Medicine, Chulalongkorn University, Bangkok, Thailand; 4 Excellence Center for Medical Genetics, King Chulalongkorn Memorial Hospital, Thai Red Cross, Bangkok, Thailand; Bellvitge Biomedical Research Institute (IDIBELL), Spain

## Abstract

Congenital heart defects (CHD) occur in 40% of patients with trisomy 21, while the other 60% have a structurally normal heart. This suggests that the increased dosage of genes on chromosome 21 is a risk factor for abnormal heart development. Interaction of genes on chromosome 21 or their gene products with certain alleles of genes on other chromosomes could contribute to CHD. Here, we identified a pair of monozygotic twins with trisomy 21 but discordant for a ventricular septal defect and epilepsy. Twin-zygosity was confirmed by microsatellite genotyping. We hypothesized that some genetic differences from post-twinning mutations caused the discordant phenotypes. Thus, next generation sequencing (NGS) technologies were applied to sequence both whole genome and exome of their leukocytes. The post-analyses of the sequencing data revealed 21 putative discordant exonic variants between the twins from either genome or exome data. However, of the 15 variants chosen for validation with conventional Sanger sequencing, these candidate variants showed no differences in both twins. The fact that no discordant DNA variants were found suggests that sequence differences of DNA from leukocytes of monozygotic twins might be extremely rare. It also emphasizes the limitation of the current NGS technology in identifying causative genes for discordant phenotypes in monozygotic twins.

## Introduction

Down syndrome (DS; OMIM 190685) is a human chromosomal disorder caused by an extra copy of genomic region on chromosome 21. It is one of the most common causes of human genetic disorders occurring at approximately 1 in 750 live births [1]. The extra copy of chromosome 21 is largely resulted from the failure of normal chromosomal segregation in maternal meiosis, which accounts for approximately 87% of DS patients [2]. Common characteristic facial features include oblique eyes, flat nasal bridge, epicanthus, and protruding tongue. Other phenotypes include intellectual deficit, hypotonia and other associated developmental disorders and congenital anomalies [3].

One of the severe phenotypes is congenital heart defect (CHD) occurring in approximately 40% of patients with trisomy 21, albeit about 0.8% occurrence in the general population [1]. This suggests that the increased dosage of genes on chromosome 21 is a risk factor but not sufficient for abnormal heart development [4]. Interaction of genes on chromosome 21 or their gene products with certain alleles of genes on other chromosomes could contribute to CHD.

In view of genetic disorders, phenotypic discordance in monozygotic twins may provide a better understanding of relevant factors that are involved in disease etiology [5]. Although, monozygotic twins are generally considered to be genetically identical, the underlying genetic differences may arise during embryonic development, for example, single nucleotide mutations, deletions, conversion, copy number variation and postzygotic mitotic recombination. These variations have been suggested as possible genetic mechanisms causing discordant monozygotic twins [6]. Some recent studies described single nucleotide polymorphism differences between monozygotic twins [7].

The detection of genetic differences generally relies on genome comparison between a sequence of interest and the reference sequence. However, in the case of monozygotic twins, their sequences can be compared against each other. Identified mismatches could be selected for validation as possible mutations causing the discordant phenotype. For instance, discordance of monozygotic twins with autosomal dominant neurofibromatosis type 1 (NF1) was explained by the presence of a *de novo NF1* mutation in all investigated cells of the affected twin, while the cells from the unaffected twin were mosaic [7].

Since only a small number of genetic differences between twins are expected [8], a highly sensitive method with high resolution and whole genome coverage should ideally be applied [9]. With the advent of next generation sequencing (NGS) technology, whole genome single nucleotide differences can efficiently be mapped [10]. Moreover, a genome-wide coverage would allow for a non-biased approach, not restricted to certain pre-selected regions. A conventional Sanger sequencing approach can be used to validate the candidate discordant variants obtained from NGS [11].

Here, we identified a pair of Thai monozygotic twin boys with trisomy 21 discordant for a CHD and epilepsy. We hypothesized that some genetic differences from post-twinning mutations, e.g., Single Nucleotide Variants (SNVs) or small insertions or deletions (Indels) caused the discordant phenotypes. Therefore, NGS was used in order to identify such genetic differences.

## Materials and Methods

### Ethical statement

The study was approved by the institutional review board of Faculty of Medicine of Chulalongkorn University. Written informed consent was obtained from the parents of patients included in the study.

### Patients

Thai twin boys born at the King Chulalongkorn Memorial Hospital, Bangkok, had hypotonia, low-set ears, upslant eyes and flat nasal bridge. A clinical diagnosis of Down syndrome was given and later confirmed by chromosome analysis indicating three copies of chromosome 21 in both twins. Here we assigned twin A as the older brother and twin B as the younger one. Echocardiography revealed that twin A had a ventricular septal defect (VSD), requiring a corrective operation when he was one year and two months old. Although twin B had a normal heart, he developed seizure when he was six months old. Even with several antiepileptics, he continued to seize until he was given vigabatrin when he was one year old; the seizure then stopped. Such seizure was not observed in twin A.

### Zygosity analysis

After informed consent, six milliliters of peripheral blood was obtained from both twins and their mother. Genomic DNA was isolated from their white blood cells using QIAamp DNA blood mini kit according to the manufacturer's instruction (Qiagen, Valencia, CA). To confirm that both twins were monozygotic, we used 13 microsatellite markers on 13 different chromosomes, from ABI PRISM Linkage Mapping Set: D1S2785, D2S206, D4S424, D5S408, D7S657, D9S164, D11S1314, D14S74, D15S127, D16S515, D18S1161, D20S117, and D21S1914. In each reaction, we used 1.2 µl of genomic DNA, 9.0 µl of True AllelePCR Premix, 1.0 µl of each primer pair in a total volume of 15 µl and performed PCR following the manufacturer's instruction. Using fluorescently labeled selective primers, DNA analysis was performed on an ABI Prism 3100 genetic analyzer (Applied Biosystems, Foster City) with GeneMapper software (Applied Biosystems).

### Genome sequencing and targeted capture exome sequencing

Genomic DNA from both twins was sent for whole genome sequencing (WGS) using the service offered by Beijing Genomic Institute (BGI), China. The sequencing was performed using Applied BiosystemSOLiD4.0 (Sequencing by Oligonucleotide Ligation and Detection) system. The primary sequencing data were analyzed by using standard SOLiD analysis workflow. After that, the sequencing reads were aligned to human genome reference sequence (UCSC hg18) using BioScope software.

Whole exome sequencing (WES) of these genomic DNAs was done using Illumina HiSeq 2000 with the service from Macrogen, Inc., South Korea. Real Time Analysis (RTA) software version 1.7 was used to perform base calling and quality scoring. The reads were then aligned to UCSC hg19 using the Burrows-Wheeler Alignment (BWA) tool [12].

### Discordant SNVs/Indels analysis

Both WGS and WES datasets of the twins were deposited to NCBI Sequence Read Archive (SRA) with the sample IDs “SAMN02680286” and “SAMN02688784” for twins A (sample name G3142) and B (G3143), respectively. The experimental IDs for WGS and WES are SRX485008 and SRX522555 for twin A and SRX487546 and SRX522556 for twin B, respectively. These samples can be downloaded from NCBI BioSample database and were registered under a project id “PRJNA240916” in the NCBI BioProject database.

Candidate single nucleotide variants (SNVs) and small insertions or deletions (Indels) were extracted by comparing the twins' alignment data (BAM files). Variant calling was done simultaneously on both alignment data from twins A and B (SAMtools mpileup) in order to avoid false positive variants. If a variant could only be observed in one twin but missing due to no or not enough coverage in the other twin, such a variant would be excluded from the candidate discordant variant set.

VarScan version 2.2.5 was used to identify SNVs from the mpileup alignment data. It compared the read counts, base quality and allele frequency between the twins. Discordant SNVs were called with the sequencing depth greater than or equal to 10X. These SNVs must be present in at least three reads with the minimum variant base quality score >15. Genome Analysis Toolkit (GATK) version 1.0.5974 was used to detect Indels using the Somatic Indel Detector command. The Indel results were compared between the two twins. If Indels were detected in only one twin, the discordant Indels would be called. These resulting discordant variants were filtered again by excluding those variants that were likely to be non-functional, e.g., synonymous variants and/or variants located outside the exonic regions.

For exome sequencing data, the alignment and variant calling were done on each twin data as a standard analysis service from Macrogen using SAMtools. We used in-house variant calling script to call SNVs and Indels. To detect discordant variants, we compared side by side at each locus of the variants. Particularly, the underlying discordance would be detected, only if enough read coverage of the corresponding variant was confirmed on both twins. Nonsynonymous variants with at least 30× coverage would be chosen. Subsequently, we picked variants with variant-supporting reads more than four reads in one twin and did not have variant-supporting reads in the other.

To screen out obvious false positive variants, each of the resulting variants from both whole genome and exome sequencing was visualized along with its alignment data from both twins using Integrative Genomics Viewer (IGV) software version 2.1. The variants that passed the aforementioned criteria would be validated.

### Discordant SNVs/Indels validation

Twins' genomic DNA extracted from leukocytes as previously described was amplified by polymerase chain reaction (PCR), using primers specific to the candidate genes that had the resulting discordant SNVs/Indels (Table S1). The PCR products were sent to Macrogen Inc., South Korea for performing Sanger sequencing. Sequences were compared between both twins to verify the discordant variants.

## Results

### Zygosity analysis

Zygosity analysis showed that both twins had the same alleles of all thirteen microsatellite markers, highly suggesting that they were monozygotic twins.

### Discordant SNVs/Indels analysis

Whole genome sequencing of both twins resulted in 27.22× and 28.67× of average coverage (Table 1). The discordant SNVs and Indels from VarScan and GATK resulted in 5,701 variants. After excluding non-exonic and synonymous variants, eight discordant variants (two SNVs and six Indels) were obtained from the whole genome sequencing data (Table 2).

**Table 1:** Summary of sequencing results from whole genome and exome sequencing data.

SOLiD 4.0 whole genome sequencing	Twin A	Twin B
Size of genome (UCSC hg18)	2.8 Gb	2.8 Gb
Data mapped to genome (base pair)	78.4 Gb	82.6 Gb
Mean read depth of whole genome	27.22X	28.67X
% Coverage of target regions (>10X)	95.4%	96.3%

HiSeq 2000 whole exome sequencing	Twin A	Twin B
Size of exome (UCSC hg19)	63 Mb	63 Mb
Total yield (base pair)	10,451 Mb	8,464 Mb
On-target base pair (mapped to target regions)	3,439 Mb	2,812 Mb
Mean read depth of target regions	44.8X	36.7X
% Coverage of target regions (>10X)	83.6%	82.5%

**Table 2:** Number of discordant variants after applying different exclusion criteria for WGS and WES experiments.

WGS filtering criteria (using VarScan and GATK to detect variants)	Number of discordant variants
No filtering	5,701
Excluding non-exonic variants (NEV)	11
Excluding NEV and synonymous variants (SV)	8

WES filtering criteria (using in-house variant calling)	Number of discordant variants
No filtering	34,226
Excluding NEV	8,640
Excluding NEV with Average depth from both twins ≤30 (AD ≤30)	1,070
Excluding NEV, (AD ≤30) and SV	703
Excluding NEV, (AD ≤30), SV and both twins have depth ≤15 (BD ≤15)	302
Excluding NEV, (AD ≤30), SV, BD ≤15 and variants from <4 reads (V4)	59
Excluding NEV, (AD ≤30), SV, BD ≤15, V4 and false discordant variants eye inspected from IGV visualization (IGV)	13
Excluding NEV, (AD ≤30), SV, BD ≤15, V4, IGV and variants that have frequency <5%	7

The exome sequencing dataset had higher average coverage at 44.8× and 36.7× for twins A and B, respectively (Table 1). Exome sequencing of both twins resulted in a total of 226,983 variants prior to the discordant analysis. We selected only discordant variants that had average sequencing depths of greater than or equal to 10X, resulting in 34,226 discordant variants. After applying the stringent filtering criteria, 13 putative discordant SNVs with no Indels were chosen to be validated (Table 2). These variants from the exome dataset share no common with the discordant variants from the whole genome sequencing dataset.

### Discordant SNVs/Indels validation

**Figure 1:**
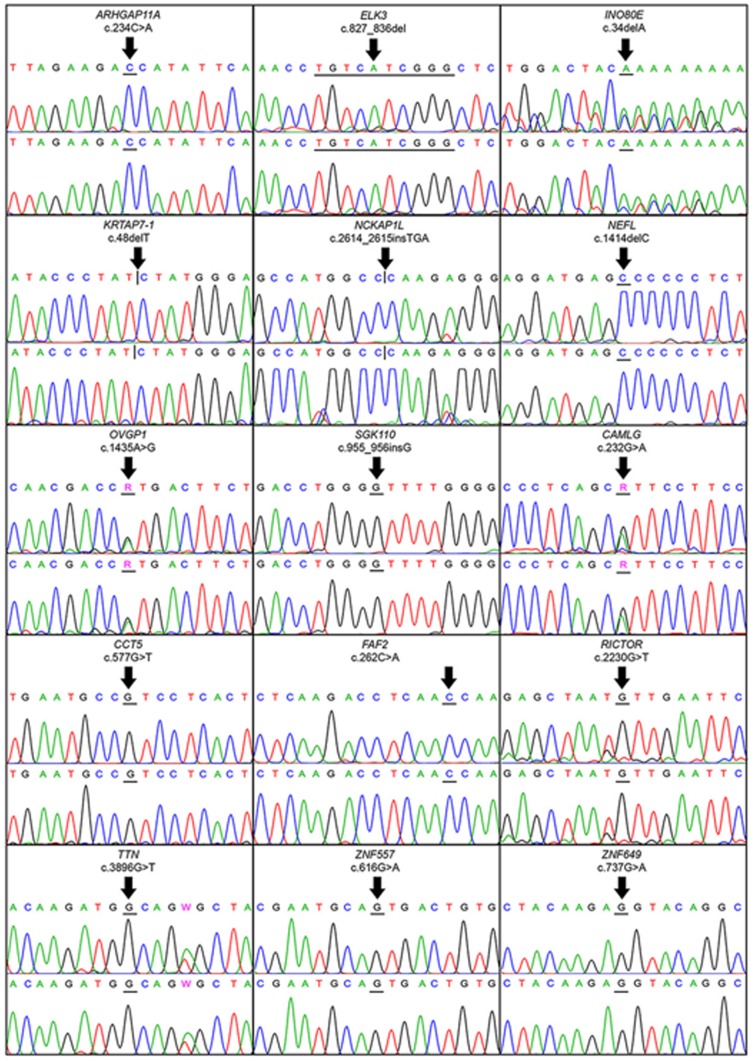
Electropherograms of Sanger sequencing. Electropherograms of Sanger sequencing of the selected 15 possible discordant variants between the two monozygotic twins identified by either whole genome or exome sequencing experiments. Upper and lower electropherograms of each panel represented twin A and twin B, respectively.

We chose 15 variants (all eight of the whole genome and seven out of 13 of the exome sequencing datasets) to be validated by conventional Sanger sequencing (Table 3). The variants from exome datasets were selected by manual observations of each variant's alignment data, using IGV to clarify the mapping result. They were selected when only two haplotypes were found, they were not located at the start or end position of reads and their base quality scores were more than 20 on average. In addition, since CHD occurring in approximately 40% of patients with trisomy 21 and the underlying variants causing discordant CHD should be common variants, we therefore selected the variants with variant frequency of not less than 5% [13]. Of the 13 discordant variants found by exome sequencing, seven met the criteria and were subjected to Sanger sequencing. Electropherograms of all 15 candidate variants showed no differences between the twins (Figure 1).

**Table 3:** Details of discordant variants from whole genome and exome sequencing data.

	Gene	Type	Chromosome	Position	Genotype		Depth of coverage Twin A		Variant Frequency Twin A	Depth of coverage Twin B		Variant Frequency Twin B	Sanger sequencing result	
					Reference	Variant	Reference	Variant		Reference	Variant		Twin A	Twin B
Genome data	*ARHGAP11A*	missense	15	30703018	C	A	34	12	26%	27	1	4%	C/C	C/C
	*OVGP1*	missense	1	111759211	A	G	11	13	54%	22	2	8%	A/G	A/G
	*ELK3*	deletion	12	95165468	TGTCATCGGG	del	8	2	20%	10	0	0%	TGTCATCGGG	TGTCATCGGG
	*NEFL*	deletion	8	24866982	C	del	6	0	0%	6	2	25%	C/C	C/C
	*NCKAP1L*	insertion	12	53211859	ref	TGA	9	0	0%	10	6	38%	ref	ref
	*INO80E*	deletion	16	29915166	A	del	11	0	0%	7	2	22%	A/A	A/A
	*SGK110*	insertion	19	60744149	ref	G	5	0	0%	5	5	50%	G/G	G/G
	*KRTAP7-1*	deletion	21	31123841	T	del	7	0	0%	4	3	43%	del	del
	*CAMLG*	missense	5	134076812	G	A	14	16	53%	1	18	95%	G/A	G/A
Exome data	*CCT5*	missense	5	10258269	G	T	31	4	11%	30	0	0%	G/G	G/G
	*FAF2*	missense	5	175913485	C	A	42	4	9%	36	0	0%	C/C	C/C
	*ZNF649*	missense	19	52394652	C	T	64	6	9%	56	0	0%	T/T	T/T
	*RICTOR*	missense	5	38958882	G	T	62	5	7%	64	0	0%	G/T	G/T
	*TTN*	missense	2	179643775	G	T	52	4	7%	49	0	0%	G/G	G/G
	*ZNF557*	missense	19	7083178	G	A	69	5	7%	61	0	0%	G/G	G/G

## Discussion

In this study, we used NGS to sequence genome and exome of the monozygotic twins with trisomy 21, discordant for VSD and epilepsy. A rigorous discordant screening revealed 15 SNVs and 6 Indels potentially causing the twin discordance. However, validation of these 15 variants via Sanger sequencing of the corresponding genes showed no differences between the twins.

Because only non-synonymous discordant variants in the coding regions were investigated, it is possible that one of the 5,690 discordant variants in the non-coding regions identified by genome sequencing [14] or one of the 367 synonymous coding variants identified by exome sequencing [15] could have functional consequences and contribute to the discordant phenotype.

Such negative results support the notion that genetic differences between monozygotic twins even with discordant phenotypes are very rare. Several previous studies also failed to map discordant SNVs in monozygotic twins with discordant phenotypes. In particular, Baranzini et al used three platforms—whole genome sequencing (WGS), duplicate array hybridization (DAH) and RNA sequencing (RNA-Seq), to identify discordant SNVs for monozygotic twins discordant for multiple sclerosis. In their study, 3,241, 126, and 322 discordant SNVs were found by WGS, DAH, and RNA-Seq, respectively. Interestingly, they found that no discordant SNVs inferred by one approach were replicated by a second approach, while 98% of concordant SNVs could be replicated by at least two methods. The validation of 15 discordant SNVs via Sanger sequencing showed identical genotypes in the twin pairs [16]. Recent studies of monozygotic twins discordant for VACTERL association, using both WES and high-density microarray approaches, also failed to identify discordant variants that could explain the discordant phenotype [17]. However, discordant variants between monozygotic twins do exist. A study of monozygotic twins discordant for schizophrenia showed two discordant SNVs which were confirmed as actual differences by Sanger sequencing [18].

NGS has successes in finding concordant variants from patients with the same disease. It has been demonstrated in a WGS study of four family members, consisting of two siblings affected with Miller syndrome and primary ciliary dyskinesia and their unaffected parents. They successfully identified the causative gene [19]. Even with a complex disorder like autism, NGS also showed some successes in identification of the causative genes. Whole exome sequencing of 16 probands revealed candidate homozygous recessive mutations in four unrelated families [20].

One possible explanation for a high false positive rate for discordant variants is that NGS technology yields high error rate results. Particularly an overall miscall error rate for Illumina platform is typically around 1% [21]. Given the human genome size of around 3 billion base pairs, it can be assumed that a genome sequencing of a person could have approximately 30 million positions of error calling. This high error rate of genome sequencing process is well illustrated by the study of monozygotic twins discordant for schizophrenia. Of the 846 discordant SNVs identified by genome sequencing, only two SNVs were confirmed as actual differences by Sanger sequencing [18].

We conducted WGS on the twins' DNA in June 2011 and found no discordant variants. Suspecting that the coverage might not be enough on the coding region, we then performed exome sequencing in June 2012, and again found no discordant variants between the two twins. We did not compare variants found by WGS and WES simultaneously. Although the alignment programs and the reference genomes (hg18 vs hg19) used for WGS and WES were different, they should not pose any problems in variant selection as we did not impose the rule that the putative discordant variants must be present in both WGS and WES.

Explanations for a pair of monozygotic twins with identical leukocytes' DNA but discordant phenotype include mosaic genomic alteration. If a somatic mutation occurs before twinning, both twins will have the mutation variant. However, they might show discordant phenotypes because the level of mosaicism in the relevant tissues of the unaffected twin does not reach the necessary level for clinical expression. In addition to mosaic state, epigenetics could be another possible explanation for the different phenotypes in monozygotic twins [22].

Of note, DNA derived from blood may not be suitable for NGS studies of discordant twins [23]. About 70% of all monozygotic twins are monochorionic and share blood circulation in pregnancies. Therefore, the hematopoietic stem cells could be transferred between them and chimeric hematopoietic systems are created. It is therefore possible that post-twinning somatic mutations in one twin could be detected in the co-twin's blood system. This will mask the underlying mutations that cause the disease in the affected twin. Therefore, it is important to sample the tissue with discordant phenotype for DNA extraction. Unfortunately, the tissues with discordant phenotypes (heart and brain) of these twins were unobtainable.

In conclusion, we applied NGS technology in monozygotic twins with trisomy 21 discordant for VSD and epilepsy. Using the stringent filtering criteria, 15 SNVs and 6 Indels were found. However, the validation of those 15 potential discordant variants via Sanger sequencing of the corresponding genes showed no differences. The false positive results emphasized the limitation of current NGS technology in identification of rare genes causing the discordant phenotypes in monozygotic twins.

## Supporting Information

Table S1 **Primer sequences used for the 15 variant validations.**
(DOC)
