# Supplementary material for: Whole Genome and Exome Sequencing of Monozygotic Twins with Trisomy 21, Discordant for a Congenital Heart Defect and Epilepsy
Source: PLoS One. 2014 Jun 20;9(6):e100191. doi: 10.1371/journal.pone.0100191 (PMC4064986; doi:10.1371/journal.pone.0100191)
Supplement: Table S1 — Primer sequences used for the 15 variant validations. (DOC) [file pone.0100191.s001.doc]

**Table S1.** Primer sequences used for the 15 variant validations

|  | Gene | Primer | Sequence 5’🡪 3’ |
| --- | --- | --- | --- |
| WGS data | *ARHGAP11A* | Forward | GGA GAA GAA TTT GGG AAG CC |
|  |  | Reverse | GTG CCA CCA CAC TCA GCT C |
|  | *OVGP1* | Forward | TTG GAG CAG GTA CTT CAG CC |
|  |  | Reverse | GTT TTG ATG CCT GCT TTG C |
|  | *ELK3* | Forward | AAC ATT TTG GTG CTT CTG CC |
|  |  | Reverse | AGG ACA ACG TTT TGA GGG AC |
|  | *NEFL* | Forward | CTG CTC CTG CTT GCC TTT G |
|  |  | Reverse | TGA GCA AGG CTT CAT TTG TC |
|  | *NCKAP1L* | Forward | CAG GGT TTT CAG GAA TCT GG |
|  |  | Reverse | GAT TCC CGC AAT ACT TCC AC |
|  | *INO80E* | Forward | TTG TAG TCC TGA CGG CAC AG |
|  |  | Reverse | ACT TGA AAG AGC CCA TGC AC |
|  | *SGK110* | Forward | GAA GGA ACC AAC TGG TGC AG |
|  |  | Reverse | TGC AAT GTG TTC CCT CTC TG |
|  | *KRTAP7-1* | Forward | GCC CAC CAG CTT GAG GTA T |
|  |  | Reverse | GCA CAT GGG AAG GTA GGA AG |
|  |  |  |  |
| WES data | *CAMLG* | Forward | TGA GTT CTA GCT GCA ACA GCA T |
|  |  | Reverse | TCT GGA AAG GTA CTG CTC TAG G |
|  | *CCT5* | Forward | AAA ATG CAG GTT GAG ATG GC |
|  |  | Reverse | TGC CTT TCT ATG CAA TTT GG |
|  | *FAF2* | Forward | CCT GTT TTG GAA TAC CAC GC |
|  |  | Reverse | AAA TAA TAC ACC CAA CGG GC |
|  | *ZNF649* | Forward | AAA CCC ATC AGC ACC AAG TC |
|  |  | Reverse | TGT TGA GAT TGC CCT TCT GA |
|  | *RICTOR* | Forward | CAG GCA TTC TTC CCT TTT CA |
|  |  | Reverse | GAG CAT GAA GAT TGG CCT AAA |
|  | *TTN* | Forward | TCA TAG CTC CTT GCC ACA GT |
|  |  | Reverse | GCC ATT TTA GCC CTC GAT TT |
|  | *ZNF557* | Forward | AAC AGA ATT CCT GGG AGA GGA |
|  |  | Reverse | TCA GAT TCG ACC TCC TCC TG |
